# Dietary total antioxidant capacity and its dietary sources in association with non-alcoholic fatty liver disease: a case–control study

**DOI:** 10.1186/s12876-026-05039-2

**Published:** 2026-06-27

**Authors:** Fatemeh Sadat Rohani, Arezoo Sadat Emrani, Bahareh Sasanfar, Fatemeh Toorang, Mahdieh Hosseinzadeh

**Affiliations:** 1 Research Center for Food Hygiene and Safety, School of Public Health, Shahid Sadoughi University of Medical Sciences, Yazd, Iran; 2 Department of Nutrition, School of Public Health, Shahid Sadoughi University of Medical Sciences, Yazd, Iran; 3 Minimally Invasive Surgery Research Center, Iran University of Medical Sciences, 13145158, Tehran, Iran; 4 Departments of Medical and Surgical Sciences, University of Bologna, Bologna, Italy; 5 Department of Nutrition, Faculty of Health, Shahid Sadoughi University of Medical Sciences, 8915173160, Yazd, Iran

**Keywords:** NAFLD, liver disease, total antioxidant capacity, vegetable oil

## Abstract

**Background:**

Total antioxidant capacity (TAC) indicates the quality of individuals’ diet. It has been proposed as a valuable measure for evaluating the potential protective effects of dietary antioxidants. The aim of this study is to assess the relationship between the dietary TAC and odds of non-alcoholic fatty liver disease (NAFLD) in Yazd, Iran.

**Methods:**

This case-control study included 115 patients diagnosed with non-alcoholic fatty liver disease (NAFLD) and 102 age- and sex-matched controls without NAFLD, all recruited from the same medical clinic. After 12 h of fasting, serum concentration of liver enzymes, fasting blood glucose and lipid profile of case and control groups were measured. To evaluate the participants’ dietary intake during the past year a semi-quantitative Food Frequency Questionnaire which was validated for Iranian population, were used.

**Results:**

There was no significant association between TAC and the risk of NAFLD in the whole population. However, risk of NAFLD in men those in top tertile of vegetable oil TAC were higher than men in the lowest tertile in crude model (OR = 3.81, CI: 1.19–12.2; *P* = 0.01). This relationship was attenuated and no longer statistically significant after adjusting for confounding variables.

**Conclusions:**

In the current study, no significant association was observed between NAFLD and dietary TAC, although in men who had a higher intake of vegetable oil TAC, a higher risk of NAFLD was observed. Further well-designed prospective studies are warranted to clarify these relationships.

**Supplementary Information:**

The online version contains supplementary material available at https://doi.org/10.1186/s12876-026-05039-2.

## Background

Today, non-alcoholic fatty liver disease (NAFLD) represents one of the most common chronic liver diseases worldwide [1]. In NAFLD, fat accumulates excessively in the liver. Initially, simple steatosis occurs; however, as the disease progress, it can cause inflammation and liver cell damage, leading to non-alcoholic steatohepatitis (NASH) and, in some cases, cirrhosis [1]. Moreover, studies indicated that NAFLD-induced mortality has increased dramatically in recent years, especially with increasing fibrosis [2, 3]. The global prevalence of NAFLD is approximately 25% [4]. One of the highest rates of NAFLD is in the Middle East, where the prevalence reaches 32% [5]. In Iran, the prevalence of this liver disease is also higher than the global average and is estimated at 35% in adults [6]. Various factors-such as obesity, insulin resistance, type 2 diabetes, hypertriglyceridemia, hypertension, low HDL-cholesterol, rapid weight loss, protein-calorie malnutrition, and the use of drugs such as glucocorticoids-have been reported as risk factors for NAFLD [7, 8]. Another critical factor in liver damage and progression of NAFLD is oxidative stress. Excessive production of reactive oxygen species (ROS) and lipid peroxidation disrupts cellular integrity in liver by damaging cellular components. Therefore, when antioxidant and anti-inflammatory defense systems become impaired, chronic steatohepatitis can develop [9].

Plant-based foods contain a variety of antioxidants, such as ascorbic acid, polyphenols, beta-carotene, and lycopene [10, 11]. Additionally, beverages like coffee, red wine, and certain foods like chocolate and whole grains are rich in antioxidants [12–14]. Studies have shown that compounds with high antioxidant content are inversely related to oxidative stress disorders [15–17]. Although the role of dietary macronutrients in NAFLD has been fully studied, the effect of micronutrients-including antioxidants-on this disease has been received less attention [18].

Total antioxidant capacity (TAC) of foods has been introduced as a tool to evaluate the positive effects of receiving antioxidants in diets and is also an indicator of the quality of an individual’s diet [19, 20]. A balanced diet plays an important role in improving NAFLD, and various studies have shown that the consumption of vegetables and fruits in the diet is inversely related to liver stiffness [21]. A case-control study was conducted to assess the relationship between dietary total antioxidant capacity (DTAC) and the odds of NAFLD. Their results indicated that a higher dietary TAC was related to a decreased risk of NAFLD. Findings from another case-control suggested that enhancing natural antioxidant capacity may help prevent the onset of NAFLD [22, 23]. However, these studies have not investigated the individual effects of the primary dietary sources of antioxidants on the development of fatty liver disease. In addition, no significant association was found between dietary intake of vitamin C and/or vitamin E and nonalcoholic steatohepatitis in a case-control study [24].

According to World Health Organization (WHO) guidelines, moderate fruit and vegetable (F&V) intake is defined as five servings of fruits and/or vegetables per day [25]. A study conducted in 2011 reported that the average daily consumption of F&V in the Iranian adult population was 2.58 servings (206 g) per day [26], which is less compared to both developed and developing countries [26]. Moreover, few studies have investigated the association between TAC and NAFLD [22, 23]. To the best of our knowledge, no study has specifically examined the relationship between NAFLD and TAC derived from antioxidant-rich food groups in Iran. Therefore, the aim of this study is to assess the relationship between the dietary TAC and TAC from food groups and risk of NAFLD in Yazd city, Iran.

## Methods

### Participant

**Fig. 1:**
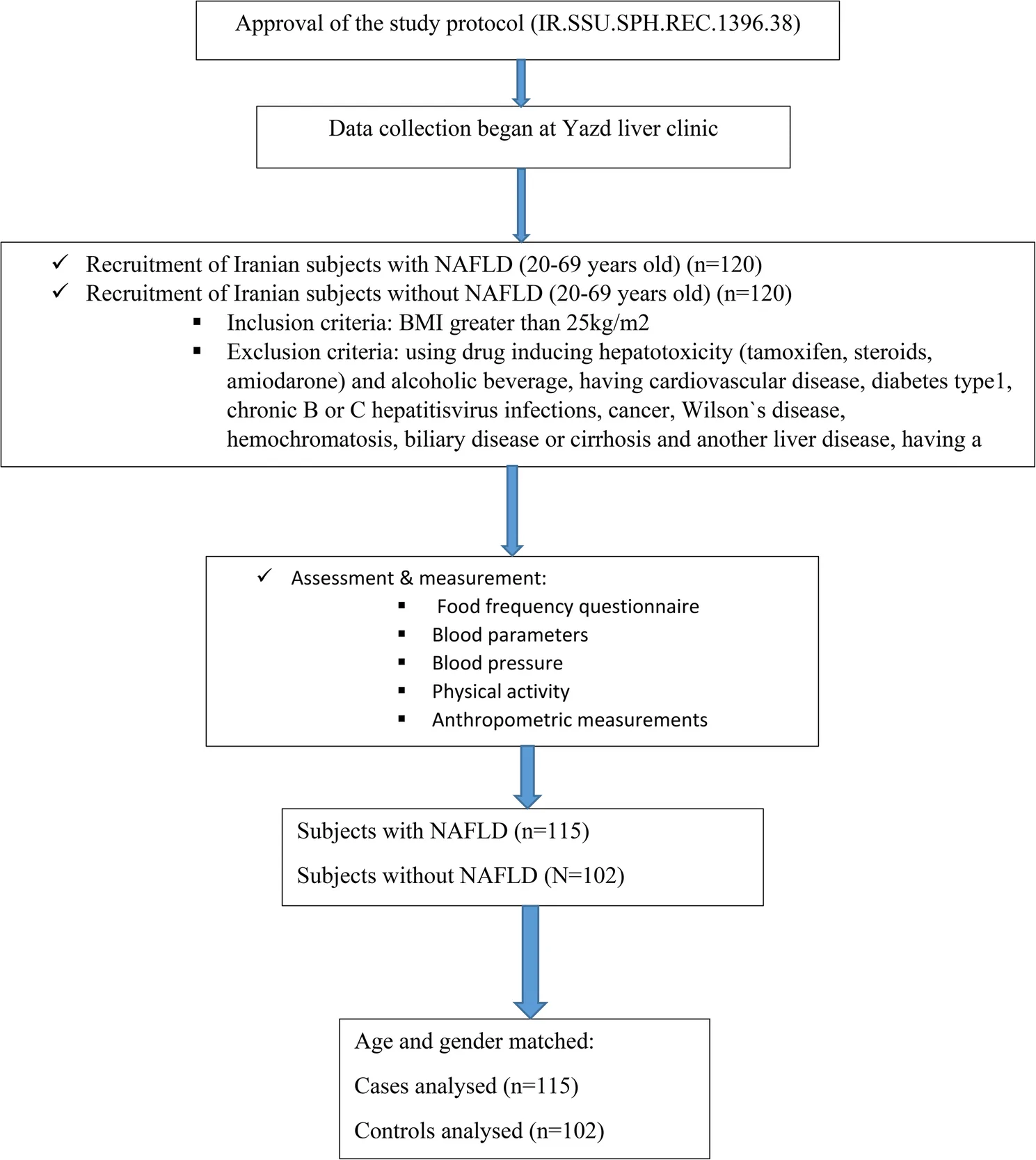
The strobe flow chart of the study. depicts enrolled, excluded, included, and final numbers for analysis

In the present case-control study, 240 individuals aged 20–69 years old were recruited. The methodology has been described in detail elsewhere [27]. In summary, case group consisted of 120 patients diagnosed with NAFLD based on laboratory tests and abdominal ultrasound. The control group included 120 participants without NAFLD, selected from the same clinic at the same time as cases, and were matched according to the age and gender (Fig. 1). The liver steatosis was estimated by abdominal ultrasound. To minimize recall bias, cases were interviewed as soon as the diagnosis was made and before they received any dietary counseling. All Participants provided written informed consent. This study was designed and reported in accordance with the STROBE (Strengthening the Reporting of Observational Studies in Epidemiology) guidelines to ensure transparency and completeness in reporting observational research.

The sample size was calculated to be 240 using a significance level of α = 0.05 with test power of 90%, based on an expected odds ratio (OR) of 1.45.

Our inclusion criteria were individuals aged 20–69 years with body mass index (BMI) greater than 25 Kg/m^2^. Exclusion criteria included: (1) use of hepatotoxic drugs or alcoholic beverages; (2) presence of disease like cardiovascular disease, diabetes type 1, chronic B or C hepatitis virus infection, Wilson’s disease, cancer, biliary disease or cirrhosis, hemochromatosis and any other liver disease and; (3) adherence to special diets such as Ketogenic, Nordic, vegetarian, etc.

After 12 h of fasting, laboratory data were collected from all participants at the beginning of the study. Serum concentration of liver enzymes-including alanine aminotransferase (ALT), aspartate aminotransferase (AST) and gamma glutamyl transferase (GGT), were measured along with fasting blood glucose and lipid profiles, including low-density lipoprotein cholesterol (LDL-c), High-density lipoprotein cholesterol (HDL-c), total cholesterol (TC), and triglyceride (TG).

This study was approved by the Ethics’ Committee of Shahid Sadoughi University of Medical Sciences, Yazd, Iran (IR.SSU.SPH.REC.1396.38).

### Dietary assessment 

A semi-quantitative Food Frequency Questionnaire (FFQ) comprising 178 items, previously validated for the Iranian population was used to assess the participants’ dietary intake during the past year [28]. Trained interviewers administered the FFQ, asking participants to report the frequency and quantity of consumption for each food item. Frequency categories included daily (once to four times, five to seven times, seven to nine times, ten times or more), weekly (once, two to four times, five to six times), and monthly (never or less than once, one to three times). To enhance accuracy in estimating portion sizes, a photo book was used as a visual reference. The FFQ also captured information on dietary supplement use. Using the Nutritionist IV software, the daily intake of nutrients and energy of each participant was calculated.

### Total antioxidant capacity (TAC) assessment

Dietary antioxidant capacity was assessed based on the impacts of food intakes on ferric reducing antioxidant potential (FRAP). The FRAP assay measures the ability of dietary antioxidants to reduce Fe3+ (ferric ion) to Fe2+ (ferrous ion). This method provides a reliable index for estimating the cumulative antioxidant effect of dietary components [29]. We considered a FRAP value for each food item in the FFQ based on their capacity to reduce ferric iron to ferrous iron using published data [30, 31]. For food items lacking specific FRAP data, the value of a comparable food was used as a proxy. TAC was computed for each individual by considering their daily food intake multiplied by the corresponding FRAP value (in mmol/100 g).

### Statistical analysis 

All statistical analyzes were perform in STATA software version 14 (STATA Corp., Lakeway Drive, USA). Dietary TAC was adjusted for total energy intake using the residual method. Then participants were categorized based on tertiles of dietary TAC. The Chi-square test and analysis of variance (ANOVA) were used to compare qualitative and quantitative variables, respectively. Logistic regression also was used to assess the relationship between the food-group–specific TAC and risk of NADLF. Multiple models were constructed to adjust for potential confounders. In the first model, adjustments were made for age and energy intake. The second model included additional adjustments for physical activity, education level, and BMI. Further adjustment for sex was performed across the total population. All analyses were done in all as well as subgroups defined by sex. In addition, given the multiple comparisons performed across food group–specific TAC variables and sex-stratified analyses, some findings may be due to chance. These results should therefore be considered exploratory and interpreted cautiously. P-value of < 0.05 was considered statistically significant.

## Results

General characteristics and dietary intakes of participants among case-control and across total antioxidant capacity tertile are presented in Table 1. Participants with NAFLD had a significantly higher BMI compared to the control group (*P* = 0.000). Moreover, individuals in the case group consumed significantly more energy (*P* = 0.004) and fruit (*P* = 0.01) than those in the control group. Participants in the highest TAC tertile had greater intakes of energy, refined grains, fruits, vegetables, high-fat dairy products, legumes, nuts, black tea, and coffee compared to those in the lowest tertile.

The association between NAFLD risk and tertiles of overall TAC, as well as TAC derived from specific food groups—including fruits, vegetables, nuts, legumes, coffee and tea, spices, and vegetable oils—was evaluated (Table 2). There was no significant association between these variables with the risk of NAFLD in the whole population. However, after stratifying by sex, in crude model, men in top tertiles of vegetable oil TAC had more risk of NAFLD compared who were in lowest tertiles, (OR = 3.81, CI: 1.19–12.2; *P* = 0.01). This association was no longer significant after adjusting for age, energy intake, physical activity, education level, and BMI.

Table 3 presents the contribution of food groups (fruits, vegetables, Nuts, Legumes, Coffee & black tea, Spices, Vegetable oil, Dairy, Cereal and Meats) to overall TAC intake (%) among cases and controls. No significant difference was observed between the groups.

**Table 1:** General characteristics and dietary intakes of participants

Variables		Cases (*n* = 115)		Controls (*n* = 102)		*P* value	dietary total antioxidant capacity tertiles						*P* value
							T1		T2		T3		
Age (year)		44.2 ± 10.3		43.5 ± 12.1		0.32	44.5 ± 12.9		44.7 ± 10.9		42.4 ± 9.44		0.4
Sex													
Female		65 (56.5)		57 (55.8)		0.92	46 (63.0)		41 (56.9)		35 (48.6)		0.21
male		50 (43.4)		45 (44.1)			27 (36.9)		31 (43.0)		37 (51.3)		
Body mass index		30.3 ± 4.1		25.2 ± 3.9		0.000	27.5 ± 5.2		28.0 ± 4.76		28.2 ± 4.31		0.65
Education													
Primary		44 (38.2)		34 (33.3)		0.17	30 (41.1)		24 (33.3)		24 (33.3)		0.73
Diploma		33 (28.7)		22 (21.5)			15 (20.5)		21 (29.1)		19 (26.3)		
University 3		38 (33.0)		46 (45.1)			28 (38.3)		27 (37.5)		29 (40.2)		
Physical activity													
Yes		67 (58.2)		70 (68.6)		0.41	49 (67.1)		41 (56.9)		47 (65.2)		0.4
No		48 (41.7)		32 (31.3)			24 (32.8)		31 (43.0)		25 (34.7)		
Energy		2283.5 ± 735.8		2018.4 ± 728.9		0.004	1718.0 ± 603.1		2140.5 ± 585.0		2704.9 ± 677.1		< 0.001
Food groups													
Refined grains (g/d)		302.5 ± 157.0		282.0 ± 155.3		0.16	248.3 ± 138.7		311.0 ± 166.5		319.8 ± 154.6		0.01
Whole grains (g/d)		8.5 ± 13.0		8.2 ± 15.6		0.42	6.56 ± 9.89		8.44 ± 15.7		10.3 ± 16.3		0.28
Processed meats (g/d)		9.34 ± 15.2		8.2 ± 12.4		0.27	8.27 ± 14.0		8.02 ± 13.7		10.1 ± 14.2		0.61
Fish (g/d)		13.5 ± 12.2		13.9 ± 29.1		0.56	9.66 ± 11.1		14.5 ± 31.6		17.0 ± 16.9		0.11
Fruits (g/d)		727.4 ± 433.7		607.6 ± 392.0		0.01	350.6 ± 209.0		633.7 ± 252.6		1033.4 ± 428.1		< 0.001
Vegetables (g/d)		285.8 ± 205.8		270.2 ± 167.1		0.27	211.6 ± 117.5		297.0 ± 169.0		365.7 ± 237.9		< 0.001
High fat dairy (g/d)		56.4 ± 84.4		57.1 ± 77.2		0.47	27.1 ± 28.0		54.4 ± 77.4		89.0 ± 105.6		< 0.001
Legumes (g/d)		32.9 ± 24.4		29.1 ± 19.3		0.1	23.9 ± 13.1		32.6 ± 22.3		37.0 ± 27.1		0.001
Nuts (g/d)		14.7 ± 15.8		15.2 ± 16.7		0.4	9.56 ± 11.0		13.0 ± 12.5		22.4 ± 20.7		< 0.001
Black tea (g/d)		258.1 ± 164.4		239.1 ± 197.6		0.21	168.5 ± 112.2		255.8 ± 160.7		324.4 ± 219.4		< 0.001
Coffee (g/d)		57.7 ± 34.6		55.5 ± 49.9		0.35	36.1 ± 23.2		56.3 ± 30.6		77.9 ± 55.6		< 0.001
DTAC, mmol /100 g		9.6 ± 4.43		8.49 ± 4.20		0.06	5.02 ± 1.26		8.27 ± 0.94		13.9 ± 3.62		< 0.001

Data are presented as *n* (%) for categorical variables and mean ± SD for continuous variables. χ2 Test for ordinal qualitative variables, ANOVA and t-test for continuous variables

*DTAC * dietary total antioxidant capacity

**Table 2:** Risk for fatty liver according to tertile of the total antioxidant capacity (TAC) with stratification by sex

		OR (95% CI)		OR (95% CI)		OR (95% CI)		
		T1		T2		T3		*P*_Trend_^a^
		**Total participants**						
Total TAC								
No.of cases/controls (115/102)		35/38		37/35		43/29		
Crude		1		1.14 (0.59–2.20)		1.60 (0.83–3.10)		0.15
Model 1		1		0.90 (0.45–1.80)		0.97 (0.43–2.14)		0.93
Model 2		1		0.85 (0.37–1.96)		1.04 (0.41–2.62)		0.92
Model 3		1		0.83 (0.36–1.92)		0.97 (0.38–2.47)		0.94
Fruit TAC		35/38		36/36		44/28		
No.of cases/controls (115/102)								
Crude		1		1.08 (0.56–2.08)		1.70 (0.88–3.30)		0.11
Model 1		1		0.84 (0.41–1.68)		1.15 (0.55–2.43)		0.68
Model 2		1		0.74 (0.31–1.75)		1.15 (0.48–2.76)		0.69
Model 3		1		0.73 (0.31–1.73)		1.08 (0.44–2.61)		0.81
Vegetable TAC								
No.of cases/controls (115/102)		38/35		35/37		42/30		
Crude		1		0.87 (0.45–1.67)		1.28 (0.66–2.48)		0.45
Model 1		1		0.75 (0.38–1.48)		0.88 (0.42–1.81)		0.71
Model 2		1		0.85 (0.38–1.91)		0.63 (0.25–1.55)		0.31
Model 3		1		0.84 (0.37–1.91)		0.58 (0.23–1.44)		0.24
Nut TAC								
No.of cases/controls (115/102)		39/34		37/35		39/33		
Crude		1		0.92 (0.48–1.76)		1.03 (0.53–1.97)		0.93
Model 1		1		0.77 (0.39–1.53)		0.71 (0.34–1.47)		0.36
Model 2		1		0.64 (0.28–1.45)		0.93 (0.38–2.25)		0.85
Model 3		1		0.65 (0.29–1.49)		0.91 (0.37–2.24)		0.82
Legum TAC								
No.of cases/controls (115/102)		37/36		38/34		40/32		
Crude		1		1.08 (0.56–2.08)		1.21 (0.63–2.33)		0.55
Model 1		1		1.02 (0.52–1.98)		0.99 (0.50–1.97)		0.99
Model 2		1		0.93 (0.41–2.10)		0.89 (0.39–2.05)		0.8
Model 3		1		0.91 (0.40–2.06)		0.89 (0.39–2.05)		0.79
Coffee and tea TAC								
No.of cases/controls (115/102)		66/66		13/2		36/34		
Crude		1		6.5 (1.41–29.9)		1.05 (0.59–1.89)		0.67
Model 1		1		6.02 (1.29-28.0)		1.03 (0.57–1.85)		0.75
Model 2		1		3.04 (0.53–17.4)		0.74 (0.36–1.53)		0.48
Model 3		1		2.80 (0.49–15.8)		0.72 (0.34–1.50)		0.44
Spices TAC								
No.of cases/controls (115/102)								
Crude		1		1.07 (0.59–1.94)		1.43 (0.65–3.14)		0.4
Model 1		1		1.04 (0.57–1.90)		1.11 (0.49–2.53)		0.79
Model 2				1.20 (0.55–2.59)		0.67 (0.25–1.82)		0.57
Model 3				1.30 (0.59–2.84)		0.71 (0.26–1.94)		0.67
Vegetable oil TAC								
No.of cases/controls (115/102)		40/42		44/38		31/22		
Crude		1		1.21 (0.65–2.24)		1.47 (0.73–2.97)		0.26
Model 1		1		1.04 (0.55–1.99)		1.19 (0.57–2.48)		0.64
Model 2		1		1.02 (0.46–2.23)		1.00 (0.41–2.41)		0.99
Model 3		1		0.96 (0.43–2.11)		1.01 (0.41–2.47)		0.97

		Female						
Total TAC								
No.of cases/controls (65/57)		24/22		21/20		20/15		
Crude		1		0.96 (0.41–2.23)		1.22 (0.50–2.96)		0.67
Model 1		1		0.88 (0.35–2.16)		0.99 (0.32–3.01)		0.96
Model 2		1		0.90 (0.30–2.71)		1.07 (0.28–3.95)		0.94
Fruit TAC								
No.of cases/controls (65/57)		23/21		21/22		21/14		
Crude		1		0.87 (0.37–2.02)		1.36 (0.55–3.36)		0.52
Model 1		1		0.84 (0.41–1.68)		1.15 (0.55–2.43)		0.68
Model 2		1		1.02 (0.33–3.15)		1.17 (0.33–4.09)		0.8
Vegetable TAC								
No.of cases/controls (65/57)		26/19		18/24		21/14		
Crude		1		0.54 (0.23–1.28)		1.09 (0.44–2.69)		0.94
Model 1		1		0.43 (0.17–1.08)		0.83 (0.29–2.34)		0.57
Model 2		1		0.58 (0.19–1.79)		0.57 (0.15–2.21)		0.39
Nut TAC								
No.of cases/controls (65/57)		24/18		18/23		23/16		
Crude		1		0.58 (0.24–1.39)		1.07 (0.44–2.60)		0.89
Model 1		1		0.48 (0.19–1.22)		0.92 (0.33–2.59)		0.78
Model 2		1		0.42 (0.13–1.29)		1.44 (0.38–5.40)		0.74
Legum TAC								
No.of cases/controls (65/57)		23/19		20/18		22/20		
Crude		1		0.91 (0.38–2.21)		0.90 (0.38–2.14)		0.82
Model 1		1		0.90 (0.36–2.25)		0.86 (0.34–2.15)		0.75
Model 2		1		1.08 (0.35–3.28)		0.82 (0.26–2.61)		0.75
Coffee and tea TAC								
No.of cases/controls (65/57)		37/39		7/1		21/17		
Crude		1		7.37 (0.86–62.9)		1.30 (0.59–2.84)		0.4
Model 1		1		6.73 (0.76–58.9)		1.17 (0.51–2.63)		0.59
Model 2		1		2.04 (0.18–22.6)		0.71 (0.25-2.00)		0.55
Spices TAC								
No.of cases/controls (65/57)		21/18		34/29		10/10		
Crude		1		1.00 (0.45–2.23)		0.85 (0.29–2.52)		0.81
Model 1		1		0.97 (0.42–2.25)		0.70 (0.22–2.18)		0.59
Model 2		1		1.60 (0.50–5.11)		0.39 (0.09–1.61)		0.26
Vegetable oil TAC								
No.of cases/controls (65/57)		29/21		19/21		17/15		
Crude		1		0.65 (0.28–1.51)		0.82 (0.33-2.00)		0.58
Model 1		1		0.62 (0.25–1.52)		0.70 (0.27–1.83)		0.42
Model 2		1		0.55 (0.18–1.68)		0.71 (0.21–2.33)		0.52

		Male						
Total TAC								
No.of cases/controls (50/45)		11/16		16/15		23/14		
Crude		1		1.55 (0.54–4.39)		2.38 (0.86–6.59)		0.09
Model 1		1		0.98 (0.30–3.13)		0.96 (0.28–3.28)		0.95
Model 2		1		0.74 (0.18–2.93)		0.76 (0.18–3.21)		0.72
Fruit TAC								
No.of cases/controls (50/45)		12/17		15/14		23/14		
Crude		1		1.51 (0.53–4.28)		2.32 (0.86–6.28)		0.09
Model 1		1		0.94 (0.29–3.08)		1.18 (0.38–3.71)		0.73
Model 2		1		0.47 (0.1–2.04)		0.82 (0.21–3.12)		0.91
Vegetable TAC								
No.of cases/controls (50/45)		12/16		17/13		21/16		
Crude		1		1.74 (0.61–4.93)		1.75 (0.64–4.71)		0.28
Model 1		1		1.25 (0.41–3.81)		1.02 (0.34–3.08)		0.98
Model 2		1		1.00 (0.27–3.68)		0.59 (0.16–2.23)		0.42
Nut TAC								
No.of cases/controls (50/45)		15/16		19/12		16/17		
Crude		1		1.68 (0.61–4.63)		1.00 (0.37–2.67)		0.98
Model 1		1		1.16 (0.38–3.50)		0.58 (0.19–1.76)		0.32
Model 2		1		0.97 (0.26–3.54)		0.56 (0.15–2.07)		0.37
Legum TAC								
No.of cases/controls (50/45)		14/17		18/16		18/12		
Crude		1		1.36 (0.51–3.62)		1.82 (0.65–5.03)		0.24
Model 1		1		0.87 (0.29–2.55)		1.08 (0.35–3.33)		0.87
Model 2		1		0.71 (0.2–2.52)		0.88 (0.24–3.22)		0.87
Coffee and tea TAC								
No.of cases/controls (50/45)		29/27		6/1		15/17		
Crude		1		5.58 (0.63–49.4)		0.82 (0.34–1.96)		0.76
Model 1		1		4.96 (0.54–45.6)		0.92 (0.36–2.34)		0.97
Model 2		1		3.39 (0.28–40.6)		0.79 (0.26–2.34)		0.74
Spices TAC								
No.of cases/controls (50/45)		20/22		18/18		12/5		
Crude		1		1.1 (0.45–2.68)		2.64 (0.79–8.82)		0.15
Model 1		1		0.85 (0.32–2.24)		1.85 (0.49–6.97)		0.52
Model 2		1		0.91 (0.28–2.98)		2.03 (0.43–9.46)		0.46
Vegetable oil TAC								
No.of cases/controls (50/45)		11/21		25/17		14/7		
Crude		1		2.80 (1.08–7.29)		3.81 (1.19–12.2)		0.01
Model 1		1		1.90 (0.68–5.28)		2.43 (0.70–8.38)		0.14
Model 2		1		1.49 (0.44–4.99)		1.57 (0.37–6.54)		0.51

Results of logistic regression analysis for the association between dietary vegetable oil TAC and NAFLD risk in men. Odds ratios (OR) and 95% confidence intervals (CI) are presented. Model 1: Adjusted for age and energy. Model 2: Adjusted for age, energy, physical activity, education and BMI. Model 3: Adjusted for age, energy, physical activity, education, BMI and sex for total participants. Variables used for stratification were not adjusted in this model

*TAC* Total Antioxidant Capacity, *OR* Odds Ratio, *CI* Confidence Interval, *T *Tertile

**Table 3:** Contribution of food groups to overall total antioxidant capacity intake (%) among participants with and without NAFLD

Group	Fruits	Vegetables	Nuts	Legumes	Coffee & black tea	Spices	Vegetable oil	Dairy	Cereal	Meats
Cases (*N* = 115)	47.8	7.16	4.07	1.23	25.1	0.55	0.24	2.20	6.80	1.45
Controls (*N* = 102)	45.7	7.48	4.54	1.21	25.8	0.53	0.25	2.24	7.12	1.55
*P*_value_	0.38	0.57	0.51	0.89	0.76	0.78	0.69	0.85	0.59	0.41

*NAFLD* non-alcoholic fatty liver

## Discussion

The present study investigated the relationship between NAFLD and TAC. Overall, we did not find a significant association between DTAC and the risk of NAFLD in the whole population or among women. However, in the crude model, a positive relationship was found between TAC derived from vegetable oil and risk of NAFLD among men. This relationship did not remain significant after adjusting for confounding variables.

There was no significant relationship between DTAC and the risk of NAFLD in this study. In our case-control analysis, no significant differences were observed in the intake of grains, meats, dairy, vegetables, nuts, legumes and coffee between the case and control groups. However, fruit intake differed significantly, with the control group consuming less fruit than the case group. Some studies suggest that fruit consumption may contribute to hepatic fat accumulation due to the higher lipogenic potential of fructose compared to glucose [25]. As a result, dietary recommendations may favor increasing antioxidant intake through vegetables rather than fruits [24]. Supporting this, a cross-sectional study conducted in China showed that green leafy vegetables intake has an inverse relationship with the prevalence of fatty liver disease [24]. This may help explain the lack of significant findings in our study.

Previous studies have highlighted the role of oxidative stress in the pathogenesis of NAFLD [32]. Research states that in individuals with metabolic syndrome or those consuming unbalanced diets, hepatic steatosis and insulin resistance often precede mitochondrial dysfunction and oxidative stress, which in turn contribute to hepatic inflammation and fibrogenesis [33]. Dietary antioxidants are known to neutralize oxygen free radicals and have been associated with the prevention of various chronic diseases [34]. Some foods and beverages, such as tea and coffee, have high dietary total antioxidant capacity. These popular beverages are rich in various polyphenolic compounds, including epigallocatechin, epicatechin gallate, caffeine, and chlorogenic acids, which are characterized by strong antioxidant properties and hepatoprotective effects [35, 36]. Contrary to our findings, a case-control study conducted in Iran on 225 patients with NAFLD and 450 healthy controls found a significant inverse association between DTAC and the odds of NAFLD [23]. Similarly, another Iranian study conducted on 200 individuals with type 2 diabetes reported that, after adjusting for confounding factors, participants in the highest tertile of DTAC had a lower risk of NAFLD compared with those in the lowest tertile [37]. Moreover, there is study on the association between DTAC and gallstones [38]. They have also suggested the relationship between reduced inflammation and disease improvement due to antioxidants [38]. However, a case-control that investigated the association between score of plant based diet and NAFLD, found no significant results [39]. Likewise, Choi et al. reported no association between vegetarian diet-typically rich of antioxidant-with NAFLD [40].

Although an initial association was observed between higher vegetable oil TAC and an increased risk of NAFLD in men, this relationship was attenuated and no longer statistically significant after adjusting for confounding variables. This attenuation, particularly in sex-stratified analyses, may be due to limited statistical power. It is important to distinguish between total antioxidant capacity (TAC) and the specific fatty acid composition of the diet. While TAC measures the overall potential of a diet to neutralize free radicals, it does not identify individual fatty acids. Vegetable oils contain various bioactive compounds and fatty acid profiles that may contribute to their hepatoprotective effects. Oils with higher proportions of MUFAs and n-3 PUFAs, as well as bioactive compounds such as tocopherols, phytosterols, oleocanthal, and sesamolin, have shown promise in the prevention of NAFLD through lipid modulation, reduction of oxidative stress, and anti-inflammatory effects [41]. Previous studies have also demonstrated the antioxidant properties of these bioactive compounds [42]. Sesame oil’s antioxidant constituents—particularly phenolic compounds and sesamin—have been shown to reduce hyperlipidemia, lipid peroxidation, and inflammation, and to provide superior hepatoprotective effects compared with sunflower oil [43]. Recent studies indicate that the diets of patients with NAFLD tend to contain higher amounts of saturated fatty acids (SFAs) and n-6 polyunsaturated fatty acids (PUFAs), along with lower levels of n-3 PUFAs [44]. The results of animal studies suggest that both the source and composition of fatty acids in the diet also may play a role in the development of NAFLD. For instance, a high-fat diet rich in omega-6 fatty acids derived from soybean oil has been shown to accelerate disease progression [45]. It is shown that PUFA from vegetable oil may contribute to fatty liver development through two proposed mechanisms: (1) Promoting hepatic cholesterol accumulation, likely by suppressing bile-mediated cholesterol excretion pathways. (2) Increasing mitochondrial damage and oxidative stress probably through lipid peroxidation [45, 46]. In this study, we could not separately assess the associations of high n-6 PUFAs, high n-3 PUFAs, and MUFAs, along with the bioactive compounds present in vegetable oils, with NAFLD, which may yield different results. Moreover, studies have demonstrated that blood antioxidant activity decrease in postmenopausal women [47]. Since we were unable to measure blood antioxidant levels or perform subgroup analyses based on menopausal status, this may help explain why the significant association was observed only in men.

### Strengths of the study

Our study is one of the few studies that explored the relationship between NAFLD and DTAC. Notably, we also assessed the relation between TAC scores derived from specific food groups-Fruit, Vegetable, Nut, Legume, Coffee and tea, Spices and Vegetable oil-with risk of NAFLD, separately, which is considered a new analysis in this field. Also, in this study, NAFLD was confirmed using abdominal ultrasound, which is more reliable than blood-based assessment.

### Limitations of the study

Several limitations should be acknowledged. Since this study has a case-control design, it is not possible to establish a causal relationship between NAFLD and DTAC. However, the observed association between vegetable oil–derived TAC and increased NAFLD risk in men may offer a valuable direction for future research. Also, misclassification bias may have occurred due to the study design. Moreover, dietary intake was assessed using a food frequency questionnaire (FFQ), which is subject to recall bias. Measurement error is a limitation in any study of diet. Moreover, the possibility of selection bias is difficult to avoid. There were potential confounders that we were unable to measure, and therefore, residual confounding might also remain even though various dietary and non-dietary confounders were adjusted. Also, many factors such as crop cultivation, storage and cooking methods can affect the antioxidant content of food which were not possible to be considered in our analysis. To compute dTAC, we applied FRAP assay, while this method might underestimate true antioxidant capacity of the diet due to not considering lipophilic antioxidants. In addition, FRAP mainly measures in vitro antioxidant activity and it might not accurately represent in vivo antioxidant activity because the bioavailability of antioxidants is highly variable [29]. For food items without any TAC value in the published papers, we used similar ones to compute FRAP score. This might further influence our finding. In addition, the low sample size limited our ability to perform subgroup analyses based on menopausal status. Given that antioxidant levels may vary between premenopausal and postmenopausal women, future studies should consider this variable.

## Conclusions

In the present study, no significant association was found between NAFLD and DTAC, although men with a higher score of TAC from vegetable oil, had a higher risk of NAFLD. Further longitudinal studies, particularly cohort designs, are needed to clarify the potential causal relationships and better understand the role of dietary antioxidants in NAFLD development.

## Supplementary Information

Supplementary Material 1.

Supplementary Material 2.

## Data Availability

The datasets used and/or analyzed during the current study are available from the corresponding authors on reasonable request.

## Abbreviations

TAC: Total antioxidant capacity
NAFLD: Non-alcoholic fatty liver disease
NASH: Non-alcoholic steatohepatitis
BMI: Body mass index
F&V: Fruit and Vegetable
ALT: Alanine aminotransferase
AST: Aspartate aminotransferase
GGT: Gamma glutamyl transferase
LDL-c: Low-density lipoprotein cholesterol
HDL-c: High-density lipoprotein cholesterol
TC: Total cholesterol
TG: Triglyceride
FRAP: Ferric reducing antioxidant potential
DTAC: Dietary total antioxidant capacity
