# Supplementary material for: Dietary total antioxidant capacity and its dietary sources in association with non-alcoholic fatty liver disease: a case–control study
Source: BMC Gastroenterol. 2026 Jun 27;26:566. doi: 10.1186/s12876-026-05039-2 (PMC13579992; doi:10.1186/s12876-026-05039-2)
Supplement: Supplementary file 1 [file 12876_2026_5039_MOESM2_ESM.docx]

**Figure 1. The Strobe Flow Chart of the Study**

Age and gender matched:

Cases analysed (n=115)

Controls analysed (n=102)

Subjects with NAFLD (n=115)

Subjects without NAFLD (N=102)

- Assessment & measurement:
  - Food frequency questionnaire
  - Blood parameters
  - Blood pressure
  - Physical activity
  - Anthropometric measurements
- Recruitment of Iranian subjects with NAFLD (20-69 years old) (n=120)
- Recruitment of Iranian subjects without NAFLD (20-69 years old) (n=120)
  - Inclusion criteria: BMI greater than 25kg/m2
  - Exclusion criteria: using drug inducing hepatotoxicity (tamoxifen, steroids, amiodarone) and alcoholic beverage, having cardiovascular disease, diabetes type1, chronic B or C hepatitisvirus infections, cancer, Wilson`s disease, hemochromatosis, biliary disease or cirrhosis and another liver disease, having a special diet

Data collection began at Yazd liver clinic

Approval of the study protocol (IR.SSU.SPH.REC.1396.38)
